# Relationship between degree of methylation of *sperm long interspersed nuclear element-1* (LINE-1) gene and alteration of sperm parameters and age: a meta-regression analysis

**DOI:** 10.1007/s10815-023-02980-z

**Published:** 2023-11-03

**Authors:** Andrea Crafa, Claudia Leanza, Rosita A. Condorelli, Sandro La Vignera, Aldo E. Calogero, Rossella Cannarella

**Affiliations:** 1 Department of Clinical and Experimental Medicine, University of Catania, Catania, Italy; 2 Glickman Urological & Kidney Institute, Cleveland Clinic Foundation, Cleveland, OH USA

**Keywords:** LINE1, Sperm epigenetics, Male infertility, Paternal age, Offspring health

## Abstract

**Introduction:**

The *long interspersed nuclear element-1* (*LINE1*) gene is a retrotransposon whose methylation status appears to play a role in spermatogenesis, the outcome of assisted reproductive techniques (ART), and even in recurrent pregnancy loss (RPL). Advanced paternal age appears associated with altered sperm parameters, RPL, poor ART outcomes, and compromised offspring health. The methylation status of *LINE1* has been reported to be affected by age. The latest meta-analysis on the *LINE1* methylation pattern in spermatozoa found no significant differences in methylation levels between infertile patients and fertile controls. However, to the best of our knowledge, no updated meta-analysis on this topic has been published recently. Furthermore, no comprehensive meta-regression analysis was performed to investigate the association between sperm *LINE1* methylation pattern and age.

**Objectives:**

To provide an updated and comprehensive systematic review and meta-analysis on sperm *LINE1* gene methylation degree in patients with abnormal sperm parameters compared to men with normal sperm parameters and to probe the association between sperm *LINE1* methylation status and age and/or sperm concentration.

**Methods:**

This meta-analysis was registered in PROSPERO (registration n. CRD42023397056). It was performed according to the MOOSE guidelines for Meta-analyses and Systematic Reviews of Observational Studies and the Preferred Reporting Items for Systematic Reviews and Meta-Analysis Protocols (PRISMA-P). Only original articles evaluating *LINE1* gene methylation in spermatozoa from patients with infertility or abnormalities in one or more sperm parameters compared to fertile or normozoospermic men were included.

**Results:**

Of 192 abstracts evaluated for eligibility, only 5 studies were included in the quantitative synthesis, involving a total of 340 patients and 150 controls. Our analysis showed no significant difference in *LINE1* gene methylation degree in patients with infertility and/or abnormal sperm parameters compared to fertile controls and/or men with normal sperm parameters, although there was significant heterogeneity across studies. No significant evidence of publication bias was found, and no study was sensitive enough to alter the results. In meta-regression analysis, we found that the results were independent of both ages and sperm concentration. A sub-analysis examining patients and controls separately was also conducted and we found a trend for a positive correlation between *LINE1* methylation and sperm concentration in the control group only.

**Conclusions:**

The results of this systematic review and meta-analysis do not suggest a determining role of sperm *LINE1* gene methylation degree in patients with infertility and/or abnormal sperm parameters. Therefore, we do not suggest including *LINE1* in the genetic panel of prospective studies aimed at identifying the most representative and cost-effective genes to be analyzed in couples undergoing ART cycles.

**Supplementary Information:**

The online version contains supplementary material available at 10.1007/s10815-023-02980-z.

## Introduction

Infertility is a widespread problem in industrialized countries, affecting about 15% of couples [1]. It is defined as the inability to achieve a pregnancy after 1–2 years of regular, unprotected sexual intercourse [2]. It may be addressed to impaired production of male or female gametes, the inability of gametes to meet or fuse, or abnormal embryo growth and development [2]. Male partners contribute to couple infertility in approximately half of the cases, and male infertility is the only cause of the problem in about 30% of cases [2].

Male infertility is usually diagnosed in the presence of abnormal parameters at the semen analysis. This can be due to numerous factors, such as testicular dysfunction, hypothalamic-pituitary disorders, seminal duct obstruction, and others [3–5]. It is important to recall that normal sperm parameters do not always translate into fertility [6], which makes it difficult to reach a diagnosis. Worryingly, despite a careful diagnostic process, the etiology of male infertility remains elusive in a significant proportion of these cases, configuring the so-called idiopathic infertility. In this regard, Tüttelmann et al. reported a causal diagnosis of infertility in only 28% of the more than 26,000 men who were referred to their center [4].

In recent years, epigenetic alterations have been suggested as a possible cause of apparently idiopathic infertility [6]. Epigenetic consists of all mitotic or meiotic molecular changes that regulate gene expression without modification of the DNA sequence [7]. In spermatozoa, the most frequent epigenetic modifications are DNA methylation, histone modification, and chromatin remodeling [6, 8, 9].

Several genes and their epigenetic status have attracted the attention of researchers for their possible involvement in reproduction. An example is the *long interspersed nuclear element-1* (*LINE1*) gene. It is one of the repetitive sequences of the human genome capable of moving to new locations. For this reason, they are also called transposable elements (TEs) [10]. In particular, TEs can be divided into transposons (which mobilize by a “cut-and-paste” mechanism) and retrotransposons (which mobilize by a TE-encoded reverse transcriptase catalyzed “copy-and-paste” mechanism). *LINE1* belongs to the latter family [10]. It represents about 17% of the human genome, so its methylation is considered an indicator of global DNA methylation status [11]. Furthermore, it is the only autonomous mobile element of the human genome [10, 12]. As a retrotransposon, it copies itself to several genomic locations by converting RNA back to DNA via reverse transcriptase using an RNA intermediate. Expression of *LINE1* results in profound changes in genome architecture and function, and also to transmobilize other TEs. A functional human *LINE1* is about 6 kb long and consists of a 5′-untranslated region (5’-UTR), which represents the internal promoter, two open reading frames (ORF1 and ORF2), and a 3′-UTR which terminates in a poly(A) tail [10]. Transcription of *LINE1* leads to the synthesis of two proteins, ORF1p and ORF2p, respectively. The former is a ~40 kDa protein with chaperon activity, while the latter one is a ~150 kDa protein with endonuclease and reverse transcriptase activities, and *LINE1* mobilization depends on this protein [10].

But what about the methylation status of *LINE1* in spermatozoa? Today, few but interesting studies suggest a possible relationship between sperm *LINE1* gene methylation rate and reproduction. Indeed, transposons play an important role in the germline, and those mechanisms that suppress their activity are crucial for transgenerational genomic integrity [13]. Methylation represents the epigenetic mechanism by which *LINE1* activity is repressed during spermatogenesis. Therefore, the alteration of its methylation rate could be a possible cause of male infertility [13].

Evidence suggests that exposure to toxic substances known to alter sperm quality, such as bisphenol A [14], alcohol, and nicotine [15], is associated with sperm *LINE1* hypermethylation. This may represent a mechanism by which these toxic substances can damage spermatogenesis. Indeed, a negative correlation has been reported between *LINE1* methylation levels and sperm motility and total motile sperm count [16]. Similarly, another study reported a negative correlation between *LINE1* methylation levels and sperm motility and sperm count in patients exposed to low doses of phthalates [17].

Impaired *LINE1* methylation appears to correlate with recurrent pregnancy loss (RPL) [18] and with the success rate of assisted reproductive techniques (ART) [6].

Interestingly, it also appears that *LINE1* methylation rate in the germline changes with age, with studies suggesting that sperm DNA methylation and *LINE1* methylation increase with age [19, 20]. This is an intriguing finding considering that, due to many factors, including socioeconomic/cultural factors and the continuous development of ART, paternal age has increased in industrialized countries. Furthermore, evidence in the literature suggests a role of paternal age in sperm quality, pregnancy outcome, impaired offspring health, and ART outcome [21–23]. Indeed, age is associated with decreased semen volume, sperm concentration, total sperm count, sperm motility, viability, worse morphology, and increased sperm DNA fragmentation [22]. Several mechanisms have been proposed to explain this association, including cellular changes, age-related decreased ability to repair cellular damage, accessory gland impairment, and structural changes in other components of the male reproductive tract [22].

Epigenetic modifications could be another mechanism of this association, since age affects all known epigenetic changes, including DNA methylation and histone modifications [24]. Furthermore, the association between paternal age and genetic disorders of the offspring has been known for decades. Evidences have recently emerged on the influence of paternal age on mental health of children with an increased risk of schizophrenia, autism, bipolar disorder, and intellectual disability [25]. Indeed, several studies present in the literature - the first dates back to 1958 [26] - have observed an association between advanced paternal age and schizophrenia, proposing epigenetic changes among the mechanisms for this association [24, 27]. Intriguingly, *LINE1* hypermethylation has been reported in association with autism spectrum disorders, schizophrenia, and mood disorders [28].

On these bases, we hypothesized that alterations in sperm *LINE1* methylation could be one of the mechanism associating paternal age with deteriorating offspring health and poor ART outcomes.

Currently, the latest meta-analysis evaluating the global sperm *LINE1* methylation status was published in 2017 [29]. The authors found no significant differences in the level of sperm *LINE1* methylation in infertile patients compared to fertile controls. However, to date, no analysis has been performed from this or any other study to investigate whether the association between paternal age and altered sperm parameters/ART failure outcome may result from an age-related increase in sperm *LINE1* gene methylation.

With these premises, the aims of this study are (1) to provide an update on the methylation status of *LINE1* gene in patients with abnormal vs. normal conventional sperm parameters and (2) to evaluate if age influences the rate of *LINE1* methylation in spermatozoa.

## Material and methods

### Search strategy

The meta-analysis was performed according to the MOOSE guidelines for Meta-analyses and Systematic Reviews of Observational Studies [30] (Supplementary table 1) and the Preferred Reporting Items for Systematic Review and Meta-Analysis Protocols (PRISMA-P) [31] (Supplementary table 2).

Articles were searched on PubMed and Scopus databases from the year of their founding until May 2023. The search strategy used the following combination of MeSH terms and keywords: “LINE1,” “gene methylation,” “fertilization rate,” “sperm DNA fragmentation,” “assisted reproductive technique,” “pregnancy rate,” “abortion,” and “miscarriage.” Additional manual searches were conducted using the relevant studies of reference lists. No language restrictions were applied in any literature search.

This meta-analysis is registered in PROSPERO with the registration no. CRD42023397056.

### Selection criteria

All eligible studies were selected following the PECOS (Population, Exposure, Comparison/Comparator, Outcomes, Study Design) model (Table 1) [32]. All observational cohort, case-control, and randomized clinical studies evaluating *LINE1* methylation levels in infertile adult men and/or with impaired conventional sperm parameters were included. The control group was represented by normozoospermic and/or fertile men. Animal studies, *in vitro* studies, reviews, meta-analyses, case reports, book chapters, and editorials were excluded. Studies in adolescents and azoospermic patients were also excluded.

**Table 1:** Inclusion and exclusion criteria according to the PECOS model [32]

	Inclusion criteria	Exclusion criteria
Population	Male patients	Adolescents, women, and patients with azoospermia
Exposure	Infertile male patients Abnormal sperm parameters (oligozoospermia and/or asthenozoospermia and/or teratozoospermia)	
Comparison	Fertile male patients Normal sperm parameters (normozoospermia)	
Outcome	*LINE1* gene methylation levels	/
Study type	Observational studies, randomized controlled studies, and case-control studies	Animal studies, *in vitro* studies, reviews, meta-analyses, case reports, book chapters, and editorials

*PECOS* Population, Exposure, Comparison/Comparator, Outcomes, Study type; *LINE1* Long interspersed element-1 gene

### Data extraction

Data on authors, year of publication, study design, type and number of cases, type and number of controls, age of cases, age of controls, sperm concentration of cases and controls, and type of sperm parameter abnormality occurring in the cases (i.e., oligozoospermia, asthenozoospermia, teratozoospermia, or a combination thereof) were extracted from the included articles (Table 2). When information was not present in the original article, the first or the corresponding authors of the original article were contacted to ask them for the missing data.

**Table 2:** Main characteristics of the population of the included studies

Author	Study design	Cases sample size	Control sample size	Mean age of cases (years) (mean ± SD)	Mean age of controls (years) (mean ± SD)	Mean sperm concentration of cases (mean ± SD)	Mean sperm concentration of controls (mean ± SD)	Outcome assessed	Method of evaluation of the outcome	LINE1 methylation (%) in cases (mean ± SD)	LINE1 methylation (%) in controls (mean ± SD)
Boissonnas et al., 2010	Cross-sectional	22 OAT	17 NZ	NA	NA	NA	118.9 ± 28.7	Methylation in LINE1 element	Bisulfite modification and PCR	44 ± 4.1	46 **±** 3.8
Dong et al., 2016	Cross-sectional	48 OZ	50 NZ	31.52 ± 3.58	32.22 ± 3.59	10.9 ± 3.86	115.98 ± 31.12	Methylation in LINE1 element	Bisulfite modification, PCR and pyrosequencing	76 ± 5.09	74.68 ± 5.18
		52 AZ	50 NZ	32.17 ± 3.27	32.22 ± 3.59	104.62 ± 29.4	115.98 ± 31.12			75.3 ± 5.1	74.68 ± 5.18
		55 TZ	50 NZ	31.13 ± 3.34	32.22 ± 3.59	111.63 ± 30	115.98 ± 31.12			74.3 ± 4.99	74.68 ± 5.18
El-Hajj et al., 2011	Cross-sectional	106 infertile	28 fertile	38.1 ± 5.62	38.33 ± 5.59	11.41 ± 5.88	56.22 ± 24.14	Methylation in LINE1 element	Bisulfite Pyrosequencing and PCR	71.2 ± 6.1	71.8 ± 5.3
Li et al., 2013	Cross-sectional	6AZ	6 NZ	NA	NA	NA	NA	Methylation in LINE1 element	Bisulfite modification and PCR	74.9 ± 3.4	79.8 ± 1.4
		6OZ	6NZ	NA	NA	NA	NA			81.1 ± 3.9	79.8 ± 1.4
Xu et al., 2016	Cross-sectional	46 AZ	49 NZ	31.95 ± 21.77	32.16 ± 22.82	43.93 ± 22.86	63.31 ± 22.89	Methylation in LINE1 element	Bisulfite conversion and MassARRAY quantitative methylation analysis	53.96 ± 4.82	54.21 ± 5.25

*AZ* asthenozoospermia, *N* normozoospermia, *NA* not available, *OAT* oligoasthenoteratozoospermia, *OZ* oligozoospermia, *SD* standard deviation, *TZ* teratozoospermia

### Quality assessment

The quality of evidence (QoE) of each study was assessed by two researchers, using the Cambridge Quality Checklists [33], which consists of three domains designed to assess the quality of studies correlates, risk factors, and random risk factors. The correlate checklist evaluates the appropriateness of sampling methods and sample size, as well as the quality of outcome and measurement of correlates, and consists of five items, each of which can be assigned a score of 0 or 1, for a total score of 5. The risk factor checklist can be rated 1, 2, or 3, respectively, if the data are cross-sectional, retrospective, or prospective, predicting higher scores for those studies with appropriate time-ordered data. The third checklist is for casual risk factors and evaluates the type of study design by assigning a score from 1 (cross-sectional study without a control group) to 7 (randomized clinical trials study).

### Statistical analysis

Statistical analysis was performed using Comprehensive Meta-Analysis Software (Version 3) (Biostat Inc., Englewood, NJ, USA) for meta-analysis of quantitative data. When comparing patients and controls, we calculated the standardized mean difference (SMD) instead of the mean difference (MD), due to the different methods by which *LINE1* gene methylation was measured in the various studies. The Cochran-*Q* and heterogeneity index (*I*^2^) were used to assess statistical heterogeneity. In particular, when *I*^2^ was less or equal to 50%, the variation of the studies was considered homogenous and the fixed effect model was used to calculate the pooled effect size. Conversely, if *I*^2^ was greater than 50%, significant heterogeneity between studies was assumed and the random effects model was adopted. Publication bias was qualitatively analyzed by the funnel plot skewness, which suggested some missing studies on one side of the graph. For quantitative analysis of publication bias, we used Egger’s intercept test, which assessed the statistical significance of publication bias. In case of publication bias, unbiased estimates were calculated using the “trim and fill” method. Furthermore, a meta-regression analysis was performed to test the effect of different parameters on *LINE1* methylation rate. Potential predictors were included as continuous variables, such as age and sperm concentration. Statistical significance was accepted for *p*-value values less than 0.05.

## Results

**Fig. 1:**
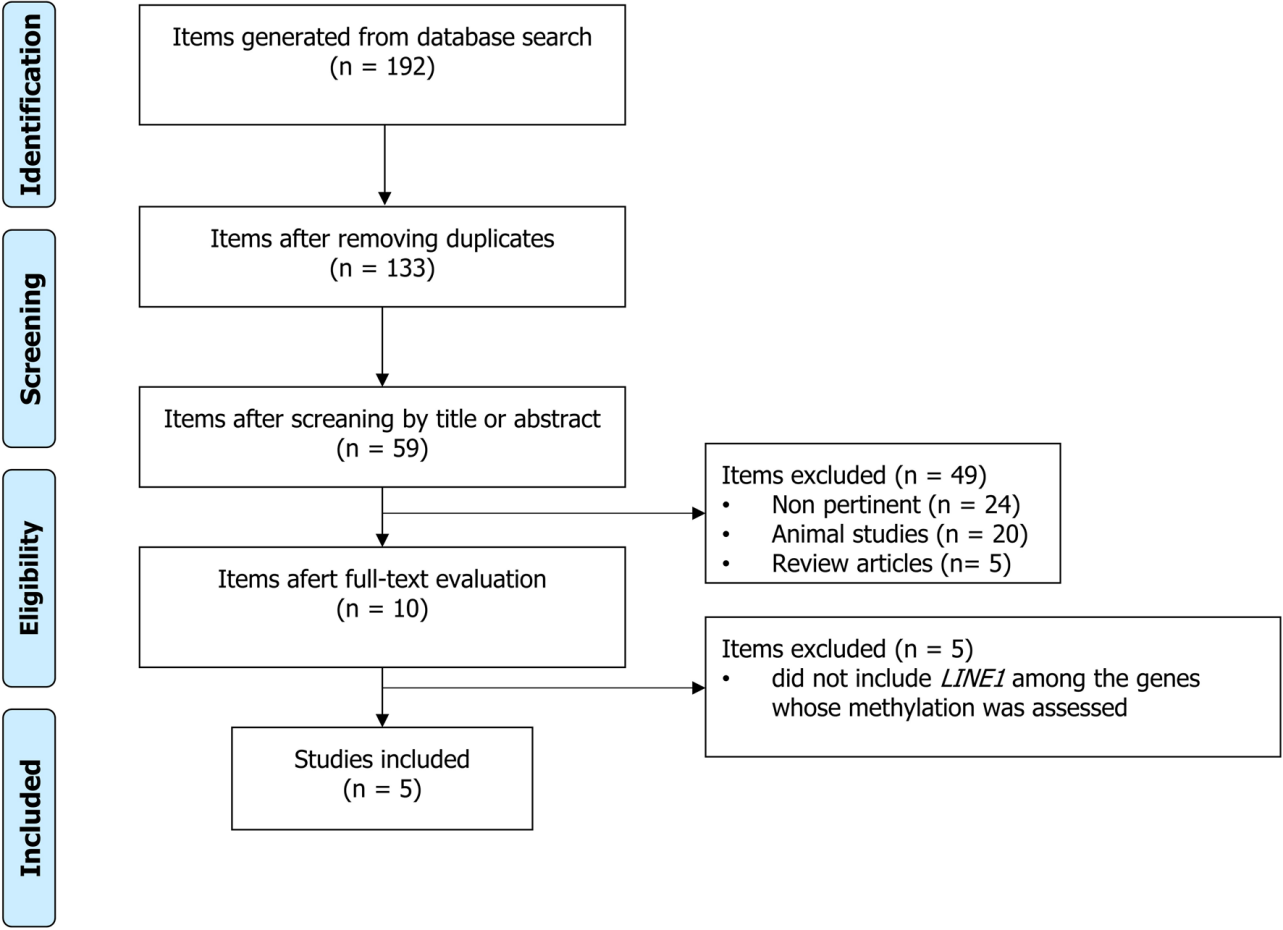
PRISMA flow-chart of the included studies

Using the above search strategy, 192 articles were retrieved. After 133 duplicate records were excluded, 59 articles were reviewed. Of these, 24 were judged not pertinent after reading their abstracts or full texts because they did not address the topic of sperm DNA methylation. In addition, 20 animal studies and 5 review articles were excluded. The remaining 10 studies were read carefully; 5 were excluded as they did not include *LINE1* among the genes whose methylation was assessed, while 5 were included in the analysis [34–38] (Fig. 1).

### Results of the QoE

All included studies were assessed using the Cambridge Quality Checklist. Although this scale does not establish a precise threshold for differentiating between high- and low-quality studies, out of a total score of 15, 4 studies scored 9 [35–38] and 1 study scored 8 [34] (Table 3).

**Table 3:** Quality of evidence assessment of the included studies [results of the Cambridge Quality Checklist].

Study name	Type of Study	Cambridge Quality Checklists		
		Checklist for correlates	Checklist for risk factors	Checklist for causal risk factors
Boissonnas et al., 2010	Cross-sectional case-control study	2	1	5
El-Hajj et al., 2011	Cross-sectional case-control study	3	1	5
Li et al., 2013	Cross-sectional case-control study	3	1	5
Dong et al., 2016	Cross-sectional case-control study	3	1	5
Xu et al., 2016	Cross-sectional case-control study	3	1	5

### Differences in patients versus controls

**Fig. 2:**
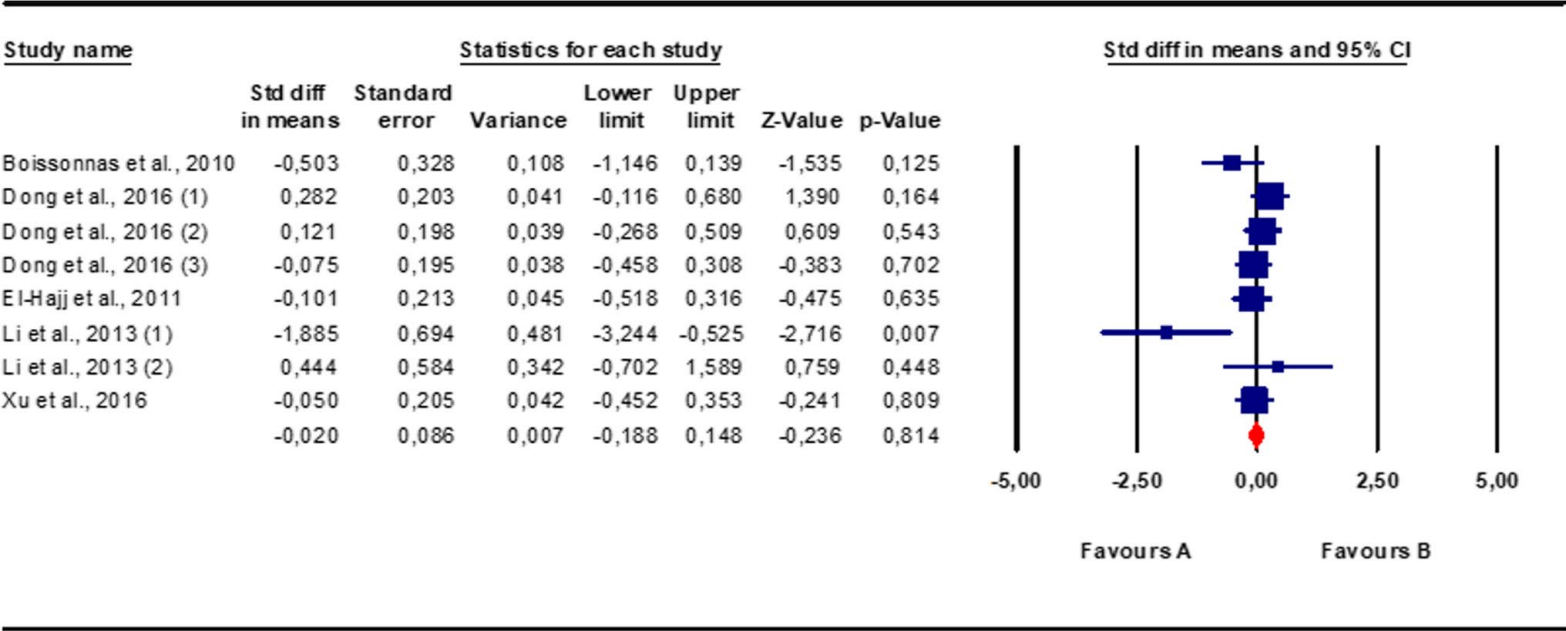
Quantitative analysis of studies that assessed the difference in LINE1 gene methylation between patients with altered sperm parameters and controls. “A” corresponds to Patients, “B” to Controls

**Fig. 3:**
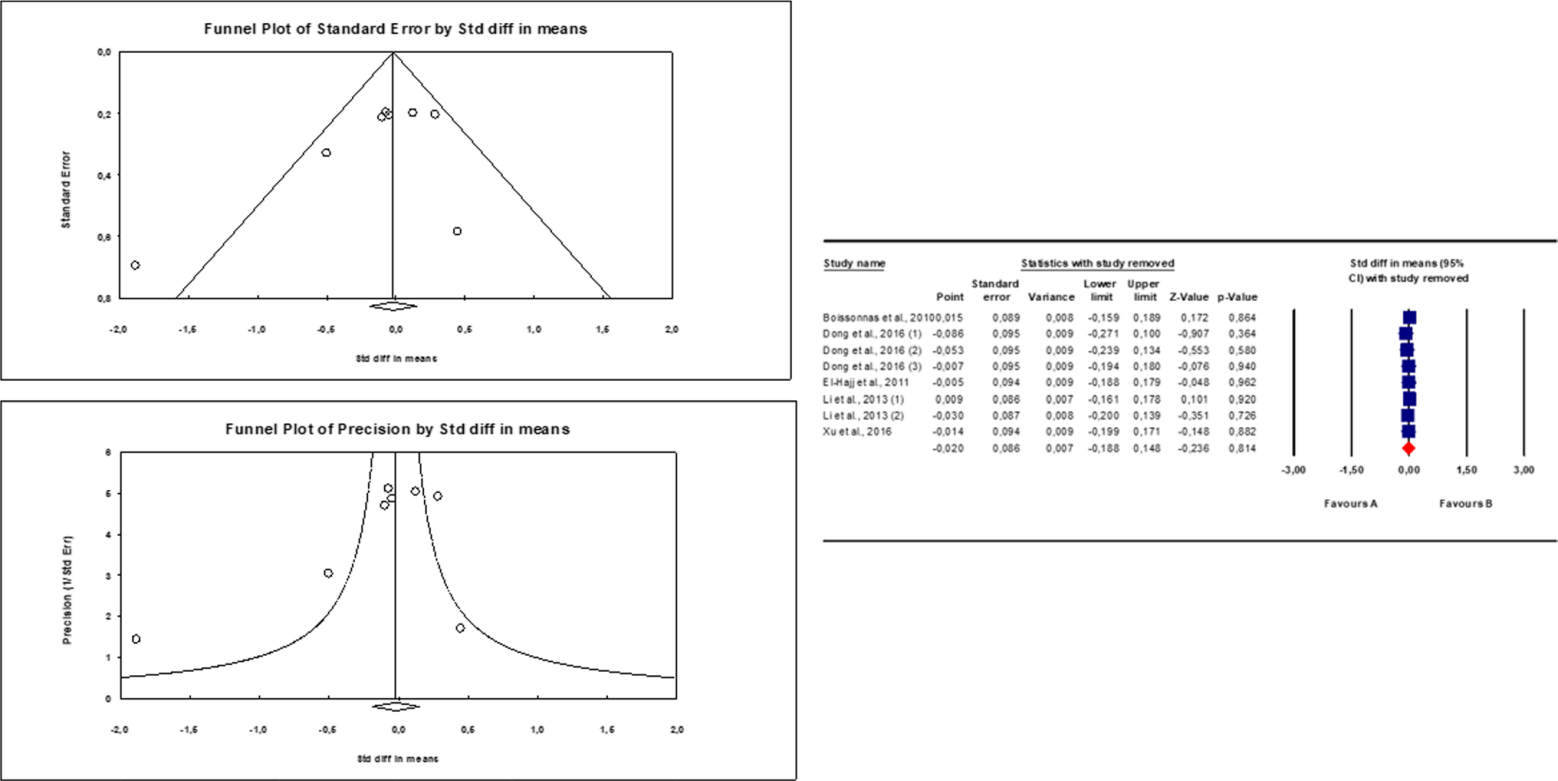
Publication bias analysis (left side) and sensitive analysis (right side) of the included studies. “A” corresponds to Patients, “B” to Controls

Because of the presence of significant inter-study heterogeneity, as shown by the Q test (*Q*-value = 12.99; *p*-value 0.07) and *I*^2^ = 46.1%, the random effect model was used. Overall, patients with infertility and/or abnormal sperm parameters did not show significantly different levels of sperm *LINE1* gene methylation compared to fertile controls and/or men with normal sperm parameters (Fig. 2). Egger’s test showed no publication bias (intercept −1.97251, 95% CI −4.98991, 1.04488, *p*=0.16081), as qualitatively highlighted also by the funnel plot, and no study was sensitive enough to bias the results (Fig. 3).

### Meta-regression analysis

#### Correlation between LINE1 gene methylation and age

**Fig. 4:**
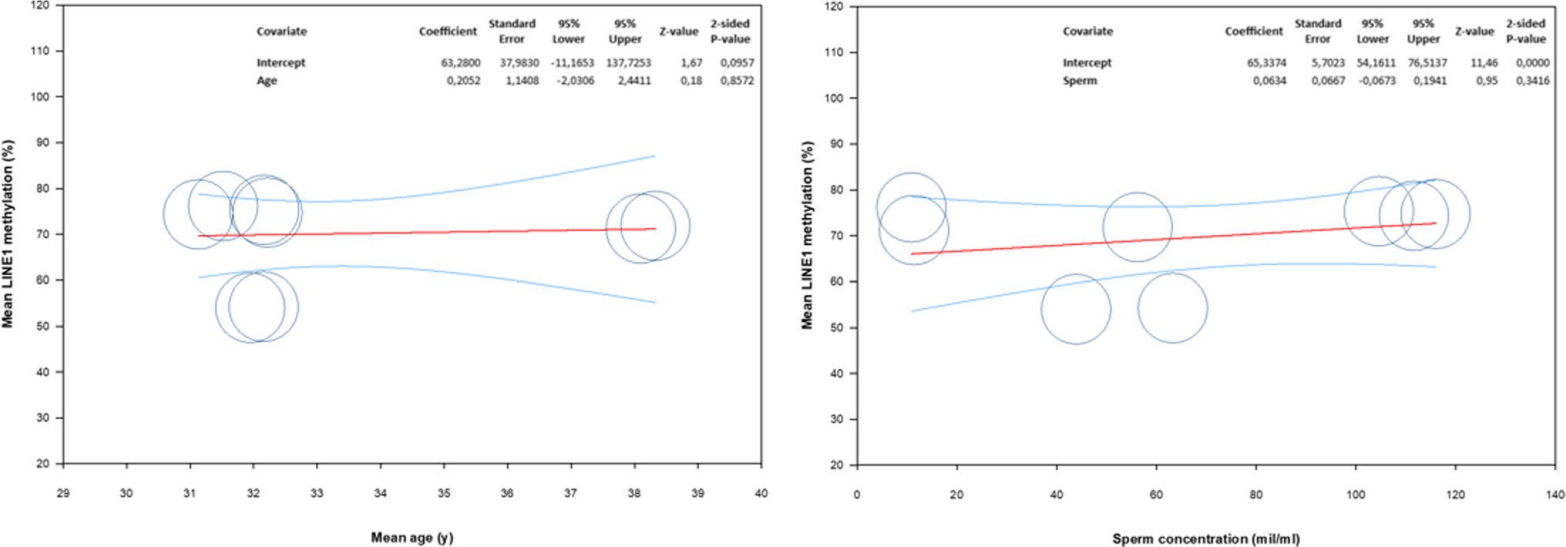
Meta-regression analysis evaluating the correlation between LINE 1 mean methylation levels and mean age (left) and sperm concentration (right) of population included

To investigate whether age affects the methylation rate of the sperm *LINE1* gene, we performed a meta-regression analysis. No correlation was found between *LINE1* methylation level and age, indicating no influence of age on *LINE1* methylation levels (Fig. 4). A sub-analysis was carried out examining patients and controls separately, with no significant correlation (patients: coefficient, 0.04; 95% CI −3.55, 3.64; *p*=0.98; controls 0.40; 95% CI: −3.22, 4.02; *p*=0.83).

#### Correlation between LINE1 gene methylation and sperm concentration

The meta-regression model we used found no significant correlation between *LINE1* methylation and sperm concentration, indicating no influence of sperm concentration on *LINE1* methylation levels (Fig. 4). A sub-analysis was carried out examining patients and controls separately, finding a trend for a positive correlation only in the control group (patients: coefficient 0.04; 95% CI −0.16, 2.24; *p*=0.72; controls 0.19; 95% CI −0.03, 0.41; *p*=0.09).

## Discussion

Due to the high prevalence of infertility and to the difficulties in identifying the etiology of male infertility in a relevant percentage of cases [4], researchers have focused on epigenetics which, in recent years, has been suggested as a possible cause of idiopathic male infertility [6]. Several genes appear to be associated with impaired sperm parameters when their epigenetics is altered and, in particular, when they are hypermethylated. Examples of these genes are *Methylenetetrahydrofolate reductase (MTHFR)*, *Paired box 8 (PAX8)*, *Neurotrophin 3 (NTF3)*, *Stratifin (SFN)*, *Harvey Rat sarcoma virus (HRAS)*, *JHM2DA*, *Insulin-like growth factor 2 (IGF2)*, *H19*, *Ras protein specific guanine nucleotide releasing factor 1 (RASGRF1)*, *Maternally expressed gene 3 (MEG3 or GTL2)*, *Pleomorphic adenoma gene 1 (PLAG1)*, *DIRAS family, GTP-binding RAS-like protein 3 (DIRAS3)*, *Potassium voltage-gated channel subfamily Q member 1 (KCNQ1)*, *Long QT Intronic Transcript 1 (LIT1)*, *Small nuclear ribonucleoprotein polypeptide N (SNRPN)*, and *Mesoderm Specific Transcript (MEST)* [8]. The spread of ART has led researchers to investigate the role of epigenetics in ART outcome and offspring health. Indeed, some studies suggest an increased risk of adverse outcomes for ART-conceived offspring compared to spontaneously conceived offspring [39]. Epigenetics has been suggested to be responsible for this, as the methylation pattern appears to be different between naturally conceived and ART-conceived offspring [40]. Two theories have been proposed to explain the higher prevalence of epigenetic aberrations in offspring conceived by ART. The same techniques, for example, could generate epigenetic alterations due to the manipulation of gametes during epigenetic reprogramming. On the other hand, these alterations could already occur in the gametes of infertile patients and, thus, transmitted to the offspring [41].

*LINE1* is a gene whose methylation status appears to play a role in reproduction. First, as a transposon, *LINE1* and those mechanisms that suppress its activity, including methylation, are crucial for transgenerational genomic integrity during spermatogenesis [13]. Furthermore, impaired *LINE1* methylation correlates with abnormal sperm parameters [16, 17], RPL [18] and with the success rate of ART [6]. Despite these premises, our meta-analysis did not find significant differences in sperm *LINE1* methylation between patients with infertility and/or abnormal sperm parameters and fertile controls and/or men with normal sperm parameters. These results are in line with a previous meta-analysis conducted on this topic [29]. Although fewer studies were included in our meta-analysis than that of Santi and colleagues [29] and the studies included were the same as those in the other meta-analysis, we decided to perform the analysis again because, during data extraction, we noticed some differences between the data from the original articles and those reported in the colleagues' meta-analysis. In case of missing data, we then contacted the authors of the original articles directly. In case of non-response, the articles were excluded, and this explains why the number of articles included in our meta-analysis is lower than that published by colleagues.

In our study, we also addressed the issue of advanced paternal age. This is because some evidence suggests an association between paternal age and sperm quality, ART success rate, RPL, and offspring health. Specifically, advanced paternal age has been associated with DNA mutations, chromosomal aneuploidies, and epigenetic changes that can be transmitted to offspring resulting in health impairments [42]. Additionally, several diseases, including autism, schizophrenia, bipolar disorders, and pediatric leukemia, have been linked to the father’s advanced age [42]. We hypothesized that the alteration of *LINE1* methylation status is a possible explanation of the above-mentioned association. Indeed, Jenkins and colleagues observed in 17 fertile men that *LINE1* methylation at the sperm DNA level, increased with age [19]. This evidence is important considering that the patients were fertile and, therefore, for the possible biological risk of the offspring born from these men [19]. Similarly, another study observed an increase in sperm *LINE1* methylation that could interfere with the developmental potential of offspring [20]. In agreement with these findings, a recently published study observed that, in contrast to other cells, telomere length in spermatozoa increases with age, and this is associated with a reduction in *LINE1* gene expression. However, the study did not analyze the methylation rate of *LINE1*, so we cannot be certain that telomere lengthening is responsible for hypermethylation and thus repression of gene expression [43]. Finally, it has recently been shown that age-related paternal hypermethylation of LINE1 can be transmitted to offspring. Indeed, a study of 141 chorionic villus samples from trisomic or with monosomy X abortions showed an increase in LINE1 methylation as paternal age increased, suggesting that LINE1 methylation may be inherited and that aging causes an increase in sperm LINE1 methylation [44].

*LINE1* hypermethylation has also been associated with neurodevelopmental disorders [28], and as previously mentioned, paternal age is associated with a higher prevalence of these disorders in offspring [42]. A study analyzing *LINE1* methylation in blood and brain tissue of mice to evaluate the dynamics of the *LINE1* gene during mammalian brain development observed methylation waves of the *LINE1* promoter in both the blood and brain during development. Blood *LINE1* methylation dynamics were similar to those observed in humans, with higher levels of methylation in the early postnatal stages and a reduction thereafter. The authors hypothesized that modulation of *LINE1* could be one of the mechanisms involved in the long-term neurodevelopment of newborns. Additionally, a post hoc analysis using Griffiths Scales demonstrated that early intervention improved neurodevelopmental outcomes of preterm infants in both the short (12 months) and long (36 months) term [45].

Conversely, a study aimed at investigating sperm DNA methylation status in couples with RPL reported a significant decrease in DNA methylation at three CpG sites in the *LINE1* promoter in the RPL group, suggesting that investigation of paternal genetic and epigenetic factors could be a useful test to identify possible causes of idiopathic RPL [18].

Based on these premises, we performed a meta-regression analysis to evaluate the relationship between sperm *LINE1* gene methylation and age. To the best of our knowledge, no meta-analytic study had previously conducted such. Reassuringly, our meta-regression results suggest no correlation between *LINE1* methylation status and age. We also carried out a sub-analysis to understand whether the relationship between *LINE1* methylation and age might differ between fertile men and infertile patients. No correlation was found in the sub-analysis, meaning that this relationship is not influenced by the fertility status. Thus, despite all the evidence reported in the literature, according to our results, *LINE1* gene does not appear to be employed in the possible mechanisms of the association between age and altered sperm parameters or between advanced paternal age and RPL or offspring health. Certainly, the main limitation of the present systematic review is the paucity and heterogeneity of the published data. This highlights the need for further studies before a definitive conclusion can be made on this matter.

## Conclusion

According to the literature, *LINE1* gene and its methylation status could play a role in spermatogenesis, pregnancy, ART success rate, and offspring health. We hypothesized that there might be a difference between patients with normal and with abnormal sperm parameters, but this hypothesis was not confirmed by the meta-analysis of the data from studies published in the literature. We also hypothesized an association between *LINE1* methylation status and paternal age, assuming that *LINE1* abnormal methylation status could explain the worse ART outcome, offspring health, and the higher rate of RPL in couples with older male partners. Reassuringly, we found no correlation between age and *LINE1* methylation status in our meta-regression analysis.

Based on these findings, it would not be worth including *LINE1* gene in the genetic panel of prospective studies aimed at identifying the most representative and cost-effective genes to analyze in couples undergoing ART cycles.

### Supplementary information

ESM 1 (DOCX 28 kb)

ESM 2 (DOCX 28 kb)
